# Photocatalytic Removal of Antibiotics on g-C_3_N_4_ Using Amorphous CuO as Cocatalysts

**DOI:** 10.3389/fchem.2021.797738

**Published:** 2021-12-08

**Authors:** Yue Zhao, Amir Zada, Yang Yang, Jing Pan, Yan Wang, Zhaoxiong Yan, Zhihua Xu, Kezhen Qi

**Affiliations:** ^1^ Key Laboratory of Optoelectronic Chemical Materials and Devices of Ministry of Education, and Hubei Key Laboratory of Industrial Fume and Pollution Control, Jianghan University, Wuhan, China; ^2^ College of Life Science, Shenyang Normal University, Shenyang, China; ^3^ Department of Chemistry, Abdul Wali Khan University Mardan, Mardan, Pakistan; ^4^ College of Physical Science and Technology, Shenyang Normal University, Shenyang, China; ^5^ Institute of Catalysis for Energy and Environment, College of Chemistry and Chemical Engineering, Shenyang Normal University, Shenyang, China

**Keywords:** amorphous CuO, cocatalysts, photocatalytic degradation, tetracycline hydrochloride, g-C_3_N_4_

## Abstract

Amorphous CuO is considered as an excellent cocatalyst, owing to its large surface area and superior conductivity compared with its crystalline counterpart. The current work demonstrates a facile method to prepare amorphous CuO, which is grown on the surface of graphitic carbon nitride (g-C_3_N_4_) and is then applied for the photocatalytic degradation of tetracycline hydrochloride. The prepared CuO/g-C_3_N_4_ composite shows higher photocatalytic activities compared with bare g-C_3_N_4_. Efficient charge transfer between g-C_3_N_4_ and CuO is confirmed by the photocurrent response spectra and photoluminescence spectra. This work provides a facile approach to prepare low-cost composites for the photocatalytic degradation of antibiotics to safeguard the environment.

## Introduction

The release of a great deal of different refractory antibiotics applied for the diseases of animal therapy, as well as for crop production into the natural water bodies by municipal and pharmaceutical industries, is considered to be a concerning issue over the past few years. The incomplete metabolism of antibiotics results in the excessive accumulation of these compounds in the ground and on the surface of water bodies to cause strong drug toxicity and reduces the efficiency of life-saving medicines. Their long persistence in ground water has led to the generation of serious antibiotic-resistant microbiota, which is a major threat to human existence. Therefore, it is extremely important to dispose these antibiotics from the bodies of ground water before they cause severe damage to human beings and aquatic animals.

Traditional methods used for removing pollutants and antibiotics are no longer effective to ensure the safety of water. Photocatalysis (Yan et al., 2020a; Yan et al., 2020b; Zhang H. et al., 2021; Li et al., 2021; Zhu et al., 2021), a green technology has recently shown exceptional performance in the photodegradation of a large number of organic pollutants (Khaledian et al., 2019; Liu et al., 2019; Yan et al., 2019; Gholami et al., 2020; Zhang et al., 2020). As a polymeric semiconductor photocatalyst, graphitic carbon nitride (g-C_3_N_4_) has shown outstanding performance and is widely favored due to its low toxicity, high chemical and thermal stability, and low-cost precursor materials (Wen et al., 2017a; Qi et al., 2020b; Di et al., 2020; Shi et al., 2020; Wu et al., 2020). Its moderate band gap and suitable conduction band (CB) and valance band (VB) positions are extremely important to produce highly active reactive oxygen species (ROS) under solar light irradiation for the effective removal of antibiotics from water (Zada et al., 2020). However, its low utilization of sunlight and high charge recombination characteristics are responsible for its low photocatalytic activity and low removal efficiency of organic pollutants (Hao et al., 2020; Wang et al., 2021). It is urgently needed to introduce the photocatalysts with improved photocatalytic activity to address water pollution.

Transition metal oxides are extremely promising materials for semiconductor photocatalysts (Zhang G. et al., 2021; Shan et al., 2021). Especially, CuO is very important due to its high abundance, low cost, non-toxic property, high intrinsic thermal safety, and environmental benignity. CuO is a p-type semiconductor with a narrow band gap between 1.2 and 1.5 eV (Liu et al., 2014). It has received much attention for its extensive applications in gas sensors, solar photovoltaics, and as a heterogeneous catalyst. It is well known that amorphous metal oxides or metal sulfides are superior to their crystalline counterparts as they possess the advantage of lower synthesis temperature, simpler synthesis process, and larger specific area. Moreover, a number of previous studies have highlighted its excellent hole/electron mobility in comparison to its crystalline counterpart, such as amorphous MoS_x_, NiS, Co_3_O_4_, etc. (Yuan et al., 2015; Wen et al., 2017b; Yu et al., 2019). Amorphous materials are considered to possess excellent redox centers in bulk on the surface to process highly efficient photocatalysis compared with its crystalline form. Although the band gap of amorphous CuO is slightly larger than that of the crystalline CuO, its excellent charge conductivity and superior surface area play an extremely positive role in the photocatalytic processes (Huang et al., 2015). Thus, it is of much interest to fabricate composite photocatalysts with amorphous CuO as cocatalyst for improving the performance of photocatalysis.

In this work, a photocatalyst composed of amorphous CuO and g-C_3_N_4_ was fabricated utilizing a facile method, which involved heating melamine in a muffle furnace and then growing different mass percent ratios of amorphous CuO. The fabricated samples showed enhanced photodegradation activities for the removal of tetracycline hydrochloride antibiotics. The improved photocatalytic performance of CuO/g-C_3_N_4_ was attributed to the extended visible light absorption and enhanced charge separation after the growth of amorphous CuO as cocatalysts on the surface of g-C_3_N_4_. Finally, a possible photocatalytic mechanism was proposed based on the experimental results. This work provides an effective method for the design of a high-efficiency photocatalyst using amorphous cocatalysts.

## Experimental Section

### Synthesis

For the preparation of g-C_3_N_4_, 20 g of melamine was ground and transferred to a 100-ml crucible. The crucible was closed and heated in a muffle furnace at 520°C for 4 h at the rate of 2°C/min. The amorphous CuO with different mass percentage ratios was grown on the surface of g-C_3_N_4_ by dispersing 0.21 g of g-C_3_N_4_ in 20 ml of solvent (10 ml of ethanol and 10 ml of water) under vigorous stirring for 30 min. A given mass of Cu(NO_3_)_2_·H_2_O was added, and stirring continued for another 30 min. Two milliliters of ammonia was added to the mixture, and the mixture was then placed in a stainless steel autoclave at 120°C for 4 h. The naturally cooled mixture was centrifuged and washed three times with distilled water to remove any impurities. It was then dried at 80°C in an oven overnight and calcined at 200°C for 1 h. The samples were represented as X%-CuO/g-C_3_N_4_ where “X%” stands for the percentage of amorphous CuO in the mixture.

### Characterization

The crystal structure of the fabricated samples was examined by x-ray diffraction (XRD) utilizing the Bruker D8 Advance Diffractometer with Cu-Kα radiation. The Fourier transform infrared (FT-IR) spectra were obtained utilizing the Nicolet Magna 560 spectrophotometer to investigate the functional groups. ESCALAB MKII x-ray photoelectron spectrometer with Mg-Kα radiation was employed to evaluate the chemical states of the elements, and the binding energies were calibrated with the binding energy of the adventitious carbon. Transmission electron microscopic (TEM) images of the samples were acquired with JEM-2010 apparatus. The ultraviolet-visible diffused reflectance spectra (UV-vis DRS) were measured with UV-3600, Shimadzu spectrophotometer with an integrating sphere attachment. The photoelectrochemical study was conducted with an IVIUM V13806 electrochemical workstation.

### Evaluation of Photocatalytic Performance

The photocatalytic activities of the different samples were evaluated from the photodegradation of tetracycline hydrochloride antibiotics. Fifty milligrams of the given antibiotic was dissolved in 1,000 ml of water. Fifty millilters of the respective solution was placed into a photochemical reactor made of quartz and mixed with 0.2 g photocatalyst. The mixture was first stirred in the dark for 30 min to attain adsorption/desorption equilibrium between the adsorbed and unadsorbed antibiotic molecules. It was then illuminated with a 500-W Xe lamp for 2 h at a wavelength of over 365 nm. After every interval of 20 min, 5 ml of the liquid portion was centrifuged, and the concentration of the antibiotics was examined with a UV-Vis spectrophotometer (UV-3600, Shimadzu) at 357-nm wavelength. The photoluminescence (PL) spectra were obtained by a Varian Cary Eclipse spectrometer.

## Results and Discussion

### X-Ray Diffraction Analysis

**FIGURE 1:**
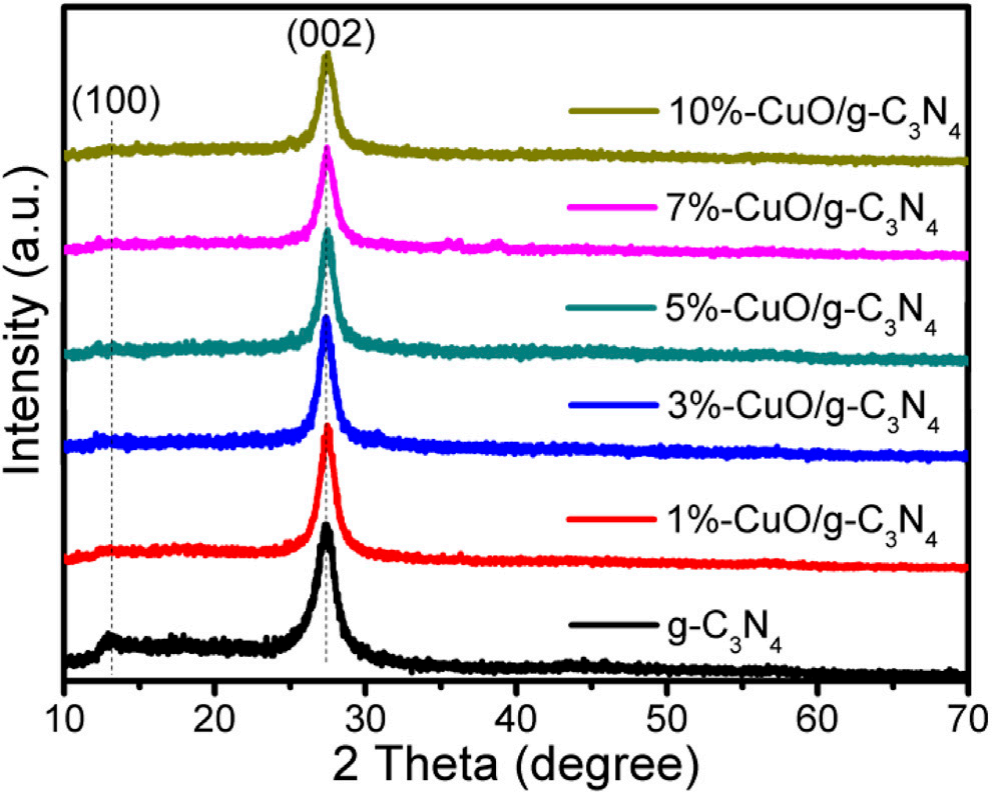
X-ray diffraction (XRD) patterns of graphitic carbon nitride (g-C_3_N_4_) and CuO/g-C_3_N_4_ samples.

The XRD patterns of the as-prepared samples are given in Figure 1. The g-C_3_N_4_ has two characteristic diffraction peaks, one at 13.02° and the other at 27.3°, which agrees well with the standard pattern of g-C_3_N_4_ (JCPDS87-1,526) (Yang et al., 2013). The peak at 13.02° is due to the inter-planar staking units of the aromatic system in conjugation, and the peak at 27.3° is due to the inter-layer structural packing units (Qi et al., 2019d; Qi et al., 2020a). These peaks are indexed to (100) and (002) crystal planes corresponding to a distance of 0.675 and 0.324 nm, respectively (Li et al., 2014; Qi et al., 2019c). After growing CuO on g-C_3_N_4_, no obvious peak of CuO was detected, indicating its amorphous nature. The intensity of the inter-planar staking peak of g-C_3_N_4_ has been reduced slightly, which shows the uniform distribution of amorphous CuO on the surface of g-C_3_N_4_.

### Ultraviolet-Visible Diffused Reflectance Spectra Analysis

**FIGURE 2:**
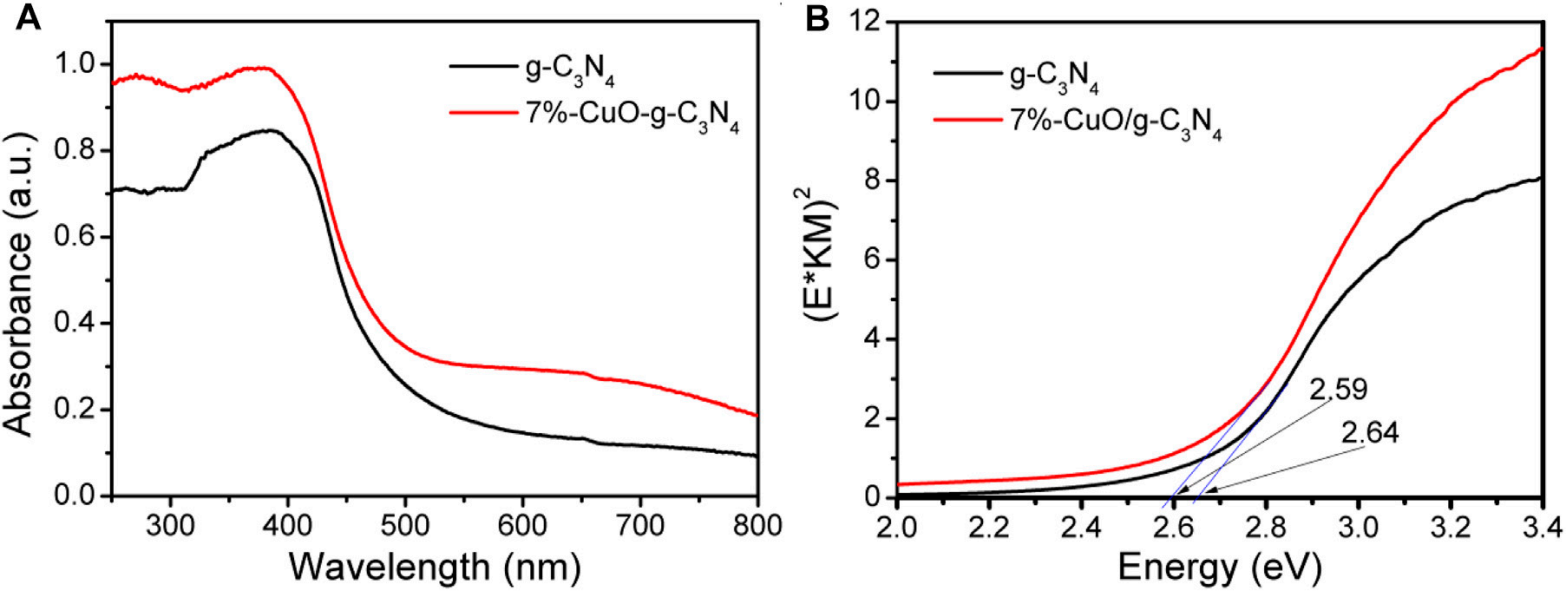
Ultraviolet-visible diffused reflectance spectra (UV-Vis DRS) of g-C_3_N_4_ and 7%-CuO/g-C_3_N_4_ samples.

The light absorption properties of the samples were investigated by UV-Vis DRS (Figure 2). The g-C_3_N_4_ absorbs the light photons with a wavelength of 470 nm and gives a direct band gap of 2.64 eV, which is consistent with the previous report (Ong et al., 2015; Qi et al., 2021). CuO has a narrow band gap of lower than 1.5 eV, which means a strong visible light absorption ability (Verma et al., 2019). When amorphous CuO was grown on the surface of g-C_3_N_4_, the light absorption was slightly increased, and the absorption wavelength was shifted slightly toward the higher wavelength side. Compared with g-C_3_N_4_, the absorption edge of 7%-CuO/g-C_3_N_4_ was shifted to 479 nm at 2.59 eV. The red shift results from the mixing of the electron orbitals of CuO and g-C_3_N_4_. The 7%-CuO/g-C_3_N_4_ sample shows a broad shoulder peak ranging from 600 to 800 nm because of the d-d transition between the energy levels of Cu2p orbital (Qi et al., 2020c). The above result concludes that CuO has been successfully loaded on the surface of g-C_3_N_4_. The light absorption property of g-C_3_N_4_ has been increased after the loading of CuO. Thus, more visible light photons can be utilized by CuO/g-C_3_N_4_ for enhanced photoactivity.

### Transmission Electron Microscopy Analysis

**FIGURE 3:**
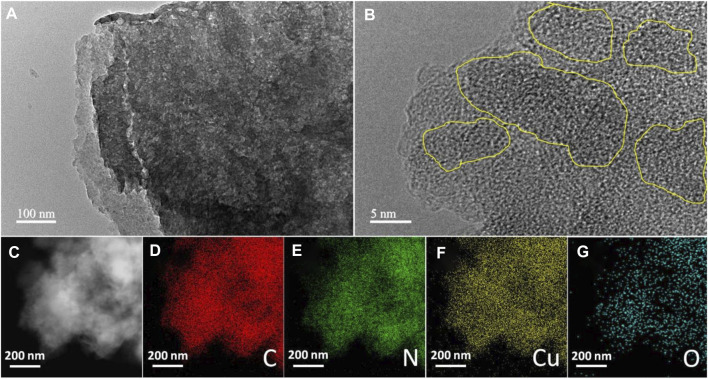
**(A)** Transmission electron microscopy (TEM) and **(B)** HRTEM images of 7-%CuO/g-C_3_N_4_, and **(C–G)** corresponding elemental mapping showing the distribution of C, N, Cu, and O.

**FIGURE 4:**
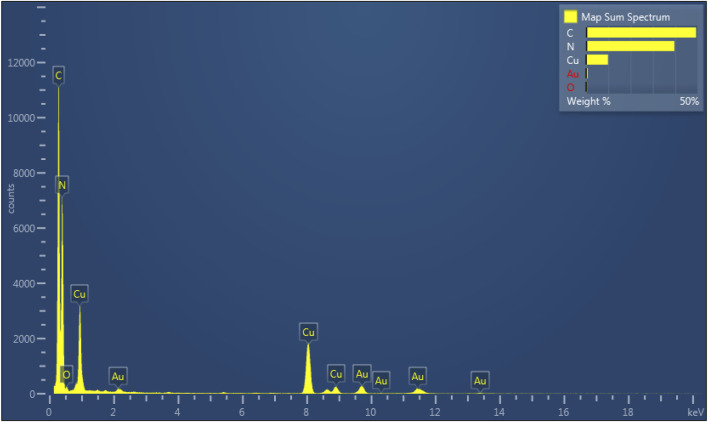
EDS spectrum of 7-%CuO/g-C_3_N_4_.

TEM measurement is an effective method to study the structural properties of 7%-CuO/g-C_3_N_4_. Figure 3A presents the TEM image of 7%-CuO/g-C_3_N_4_ with thin multilayered structures. When CuO was grown on its surface, the layered structure of g-C_3_N_4_ still persisted, and its morphology showed no detectable changes due to a low synthesis temperature approach (Figure 3B). The absence of the lattice fringes of CuO in the composite is an indication that CuO is amorphous. The elemental mapping of the sample suggests the presence of C, N, O, and Cu in the composite. The distribution of both Cu and O shows that these elements are uniformly and homogeneously grown on the surface of g-C_3_N_4_, as shown in Figures 3C–G. EDS spectrum (Figure 4) demonstrates that C, N, Cu, and O elements are evenly distributed on the 7%-CuO/g-C_3_N_4_ sample. Additionally, the existence of CuO was further verified with EDS spectra.

### Fourier Transform-Infrared Analysis

**FIGURE 5:**
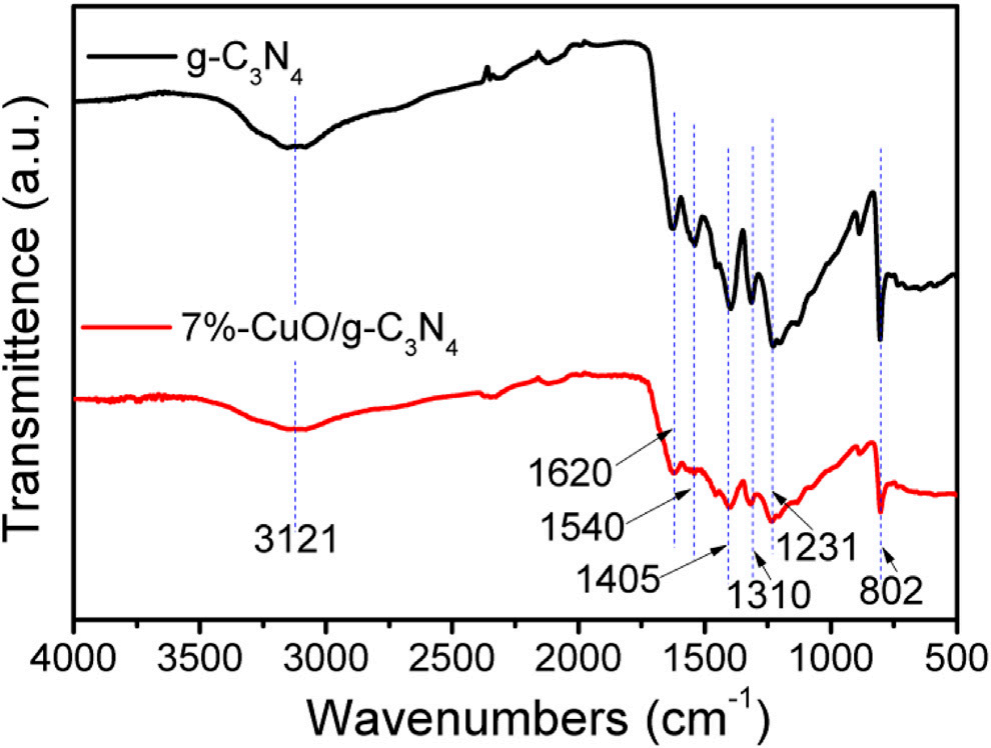
Fourier transform-infrared (FT-IR) spectra of g-C_3_N_4_ and 7%-CuO/g-C_3_N_4_ samples.

The FT-IR spectra were obtained to investigate the functional groups in the fabricated samples, and the results are shown in Figure 5. The peak at 802 cm^−1^ is attributed to the breathing mode of triazine rings of g-C_3_N_4_ (Zhu et al., 2017; Huo et al., 2019). The FT-IR peaks between 1,231 and 1,620 cm^−1^ are accredited to the C-N stretching mode of the aromatic ring, and these originate from C≡N stretching modes (Dai et al., 2014; Li et al., 2017). A broader peak at 3,121 cm^−1^ is attributed to the stretching vibrational mode of the –NH group of the aromatic ring (Cao et al., 2017; Qi et al., 2020d). These peaks are slightly reduced in their intensities when amorphous CuO was grown on g-C_3_N_4_. The FT-IR results indicate that the structure of g-C_3_N_4_ remained unchanged after the loading of CuO. This means that CuO has been well combined with g-C_3_N_4_, which is consistent with the XRD result.

**FIGURE 6:**
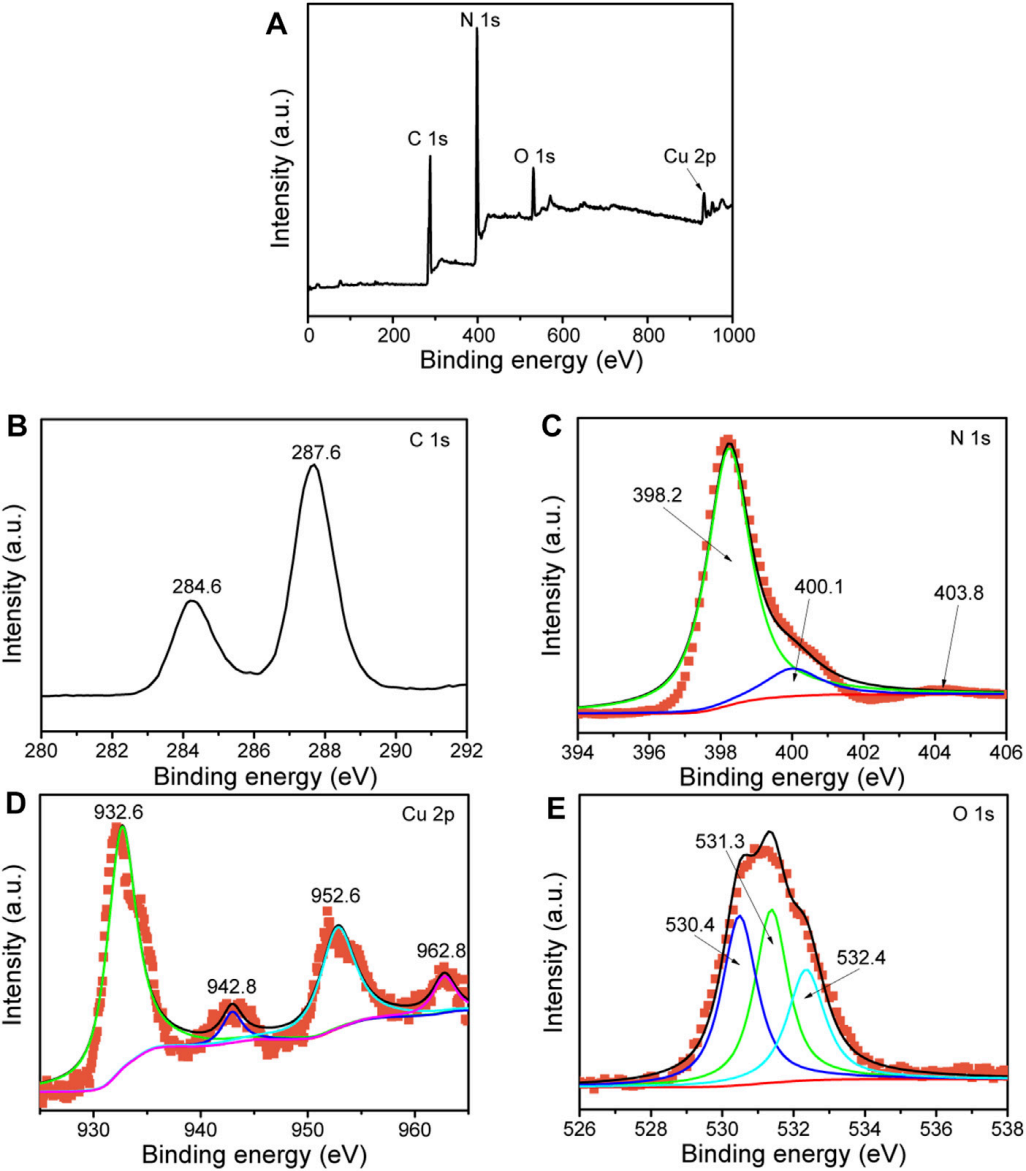
X-ray photoelectron spectroscopy (XPS) spectra of 7%-CuO/g-C_3_N_4_: survey scan **(A)**, C1s **(B)**, N1s **(C)**, Cu2p **(D),** and O1s **(E)**.

The surface chemical states of the elements in the composite were determined by XPS measurement. The XPS results demonstrate that the 7%-CuO/g-C_3_N_4_ sample contains C, N, Cu, and O elements as shown in Figure 6A. High-resolution XPS spectrum of carbon is shown in Figure 6B. Carbon shows two binding energy peaks at 284.6 and 287.6 eV, which are attributed to the sp2-hybridized C-atom bonded to the N-atom of the aromatic ring and NH_2_ group, respectively (Hayat et al., 2019; Wu et al., 2015). The binding energy peaks of the N-atom are located at 398.2, 400.1, and 403.8 eV (Figure 6C). The former two peaks are due to the C=N-C and N-(C)_3_, respectively, while the last peak is attributed to the π–π* satellite (Lin et al., 2015; Qi et al., 2019a). The binding energy peak at 932.6 eV with a satellite peak at 942.8 eV is attributed to the Cu2p_3/2_, and the peak at 952.6 eV with a satellite peak at 962.8 eV is due to the Cu2p_1/2_ of Cu2p (Figure 6D) (Shen et al., 2018). The XPS peaks of the O-atom are located at 530.4, 531.3, and 532.4 eV (Figure 6E), which are attributed to the O-atom of the crystal lattice of CuO and O-atom of adsorbed water molecules, respectively (Qi et al., 2018). The obtained XPS data indicate that CuO has been effectively coupled with g-C_3_N_4_.

### Photocatalytic Activity

**FIGURE 7:**
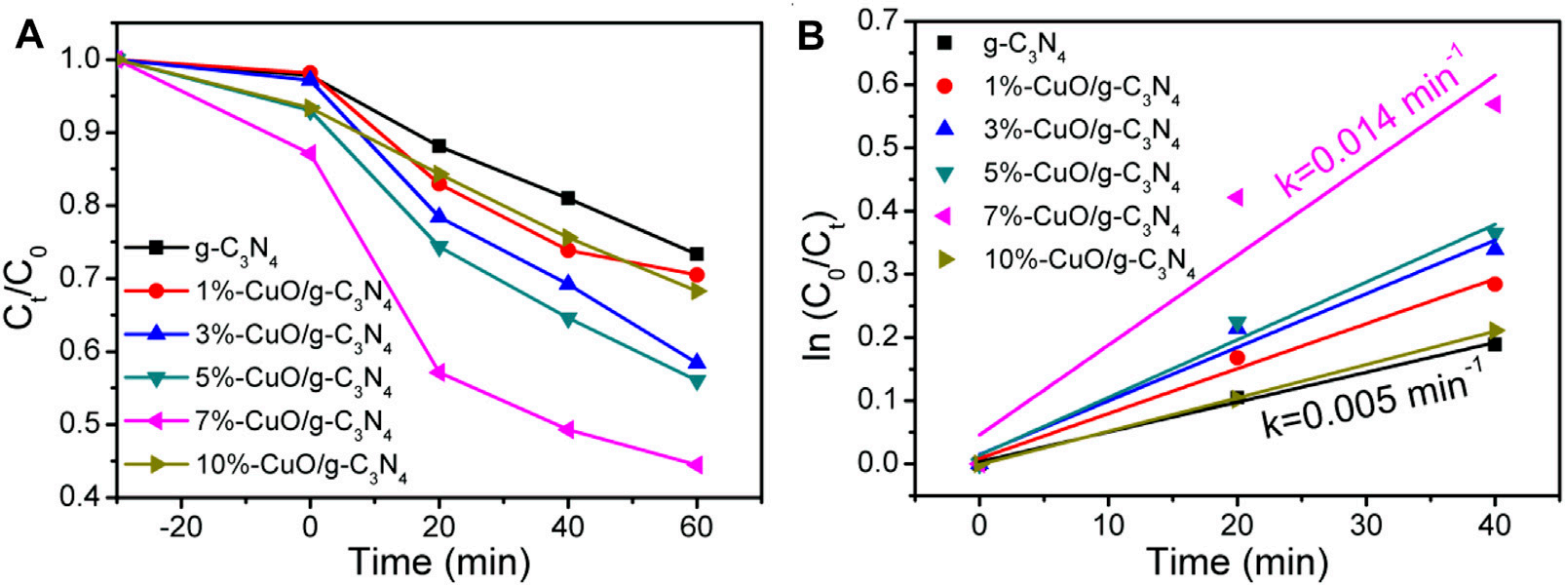
Photoactivities for the decomposition of tetracycline hydrochloride using g-C_3_N_4_ and CuO/g-C_3_N_4_ as photocatalysts.

The photocatalytic activities of the as-prepared CuO/g-C_3_N_4_ composites were evaluated by selecting tetracycline hydrochloride antibiotic for degradation under the irradiation from simulated solar light (*λ* > 365 nm). From Figure 7A, the photocatalytic activity of g-C_3_N_4_ is very low due to poor charge separation. When amorphous CuO was grown on the surface of g-C_3_N_4_, the photocatalytic activities were improved, and the degradation efficiency increased as the amount of CuO increased. After reaction for 60 min, the degradation of tetracycline hydrochloride is 24% for g-C_3_N_4_ and 55% for 7%-CuO/g-C_3_N_4_. The apparent reaction rate constant (k) is calculated in the photocatalytic degradation of tetracycline hydrochloride (Figure 7B). The kinetic constant of 7%-CuO/g-C_3_N_4_ (0.014 min^−1^) is almost three times higher than that of g-C_3_N_4_ (0.005 min^−1^). The improved photocatalytic activity is related to the extended visible light absorption and improved charge separation due to the introduction of amorphous CuO on the surface of g-C_3_N_4_. Another important role of CuO is acting as an oxidation cocatalyst, which promotes the separation and transport of holes and improves the oxidation ability of the photocatalyst.

### Electrochemical Analysis

**FIGURE 8:**
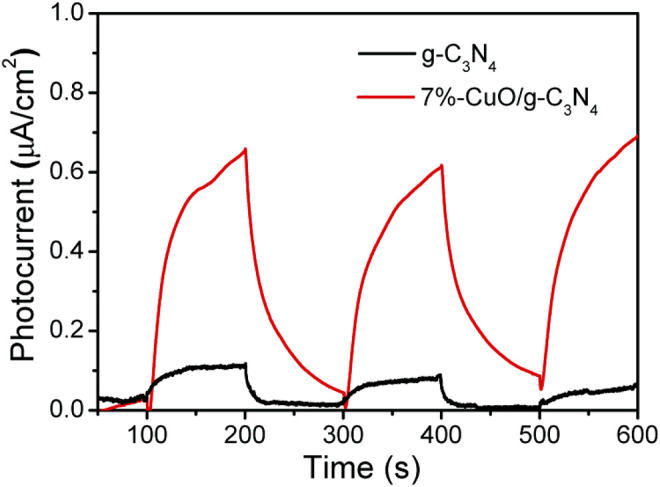
Photocurrent response signals of 7%-CuO/g-C_3_N_4_.

The measurement of transient photocurrent against time during photoelectrochemical measurement was used to study the charge separation for photocatalysis. The photocatalysts were deposited on the surface of indium-doped tin oxide glass and were used as the working electrode, while Ag/AgCl and platinum were used as the reference electrode and counter electrode, respectively. The electrolyte solution was composed of 0.1 M KCl. The greatly improved photocatalytic activity of the optimized 7%-CuO/g-C_3_N_4_ sample was attributed to the excellent charge separation in the given composite. The photocurrent response spectra were obtained, as shown in Figure 8. The 7%-CuO/g-C_3_N_4_ shows enhanced photocurrent compared with pure g-C_3_N_4_, which suggests that charge recombination has been quenched in the given sample to impart excellent photocatalytic activity. The photocurrent response spectra show that the interface of amorphous CuO and g-C_3_N_4_ favors the charge transfer and separation in the composite, indicating its important role in the photocatalytic process.

### Photoluminescence Analysis

**FIGURE 9:**
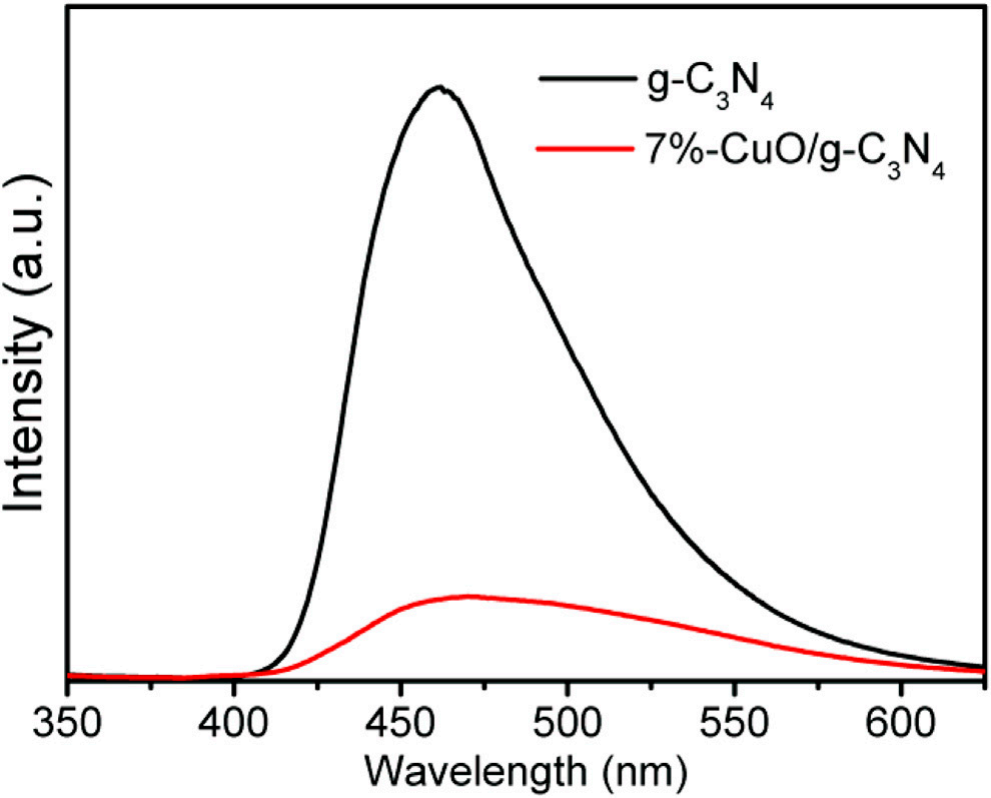
Photoluminescense (PL) spectra of g-C_3_N_4_ and 7%-CuO/g-C_3_N_4_ samples.

In order to show the enhanced charge separation in the fabricated CuO/g-C_3_N_4_ composite, PL spectra were obtained, as shown in Figure 9. As can be observed, an emission peak of g-C_3_N_4_ is located at 460 nm, which agrees with the previous results (Liu and Ma, 2020; Zhang M. et al., 2021). The intensity of PL peak is high in case of g-C_3_N_4,_ which shows poor charge separation. However, when amorphous CuO was grown over g-C_3_N_4_, the PL intensity was significantly reduced. Since the PL peak is low, charge separation is high (Lu et al., 2017; Qi et al., 2019b). The lower PL signal shown by the 7%-CuO/g-C_3_N_4_ composite is due to the adsorption of amorphous CuO on g-C_3_N_4_ surface that extends the internal charge transformation and decreases the charge recombination between the excited e^–^ and h^+^. This has increased the lifetime of working charges to result in increased reaction time. It is concluded that the optimized composite is suitable for enhancing the photocatalytic degradation of antibiotics due to an enhanced charge separation.

### Photocatalytic Mechanism

**FIGURE 10:**
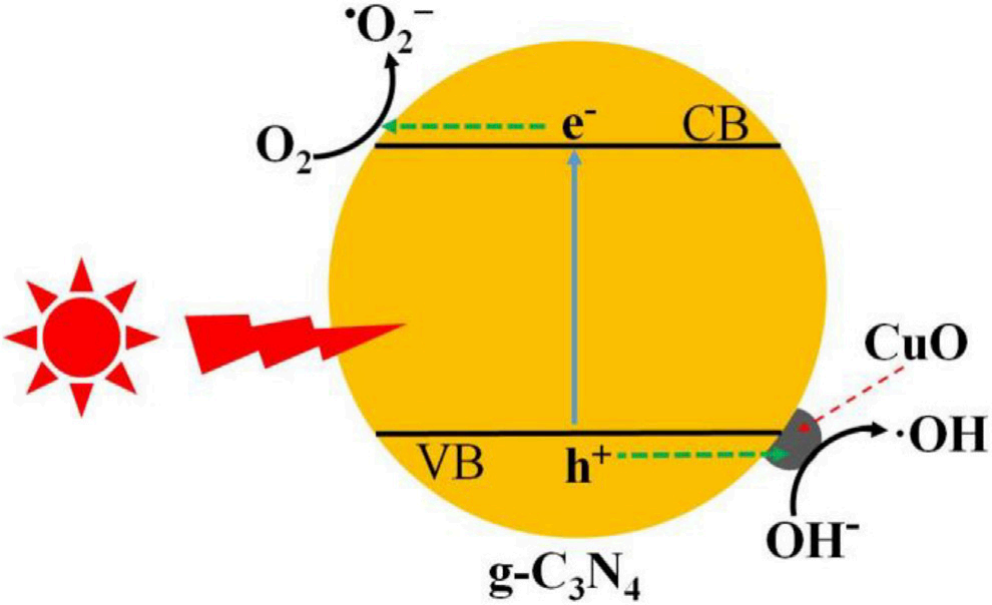
Schematic representation of charge generation and separation in CuO/g-C_3_N_4_ for the degradation of tetracycline hydrochloride.

The photocatalytic degradation of antibiotics over amorphous CuO-coupled g-C_3_N_4_ has been discussed in detail. The charge separation and transformation are illustrated in Figure 10. When irradiated with light, the electrons are promoted to the CB of g-C_3_N_4_, leaving positive holes in the VB. The generated electrons in the CB easily transfer to the g-C_3_N_4_ surface, in which the electrons can reduce the adsorbed O_2_ molecules to form super oxide anions (^•^O^2−^), which either directly reacts with antibiotic molecules. At the side of the VB, when p-type semiconductor CuO was closely attached on the n-type semiconductor of g-C_3_N_4_, the p–n junction was formed at the interface of CuO and g-C_3_N_4_ (Belaissa et al., 2016; Hua et al., 2019). Thus, the inner electric field in the p–n junction promotes the transfer of holes from the VB of g-C_3_N_4_ to CuO, and these holes oxidize the water to produce hydroxyl radicals (^•^OH) to degrade the antibiotics. Thus, the CuO/g-C_3_N_4_ composite shows an improved photocatalytic performance for the decomposition of organic pollutants.

## Conclusion

In conclusion, the amorphous CuO with excellent charge conductivity was loaded on the surface of g-C_3_N_4_ to form a composite and then applied for the photodegradation of tetracycline hydrochloride antibiotic. The prepared CuO/g-C_3_N_4_ composites show enhanced photocatalytic activities compared with the bare g-C_3_N_4_. It has been found that the g-C_3_N_4_ loaded with CuO shows the considerably positive effect for charge transfer in the composites. This work provides a facile and feasible approach for the preparation of low-cost composites for the photocatalytic degradation of antibiotics to safeguard our environment.

## Data Availability

The original contributions presented in the study are included in the article/supplementary material. Further inquiries can be directed to the corresponding authors.
